# Rare Metastatic Mesothelioma Occupying Intra-Atrial Cavity, Released by an Emergency Surgery: A Case Report and Literature Review

**DOI:** 10.70352/scrj.cr.24-0176

**Published:** 2025-02-27

**Authors:** Tomohiro Takano, Shuta Sato, Ichiro Ito, Manabu Yamamoto, Katsuaki Tsukioka, Yu Matsumura, Tetsuya Kono

**Affiliations:** 1 Division of Cardiovascular Surgery, Nagano Red Cross Hospital, Nagano, Nagano, Japan; 2 Division of Pathology, Nagano Red Cross Hospital, Nagano, Nagano, Japan; 3 Division of Respiratory Medicine, Nagano Red Cross Hospital, Nagano, Nagano, Japan

**Keywords:** cardiovascular oncologic emergencies, malignant mesothelioma, metastatic cardiac tumor, *p16/CDKN2A* codeletion

## Abstract

**INTRODUCTION:**

Cardiac surgery for cardiovascular-associated mesothelioma has a poor prognosis. However, life-saving surgery is unavoidable to maintain circulation. This report describes a case in which metastatic intracardiac mesothelioma triggered sudden respiratory failure, which was reduced by surgical resection.

**CASE PRESENTATION:**

An 81-year-old man with a history of asbestos exposure presented to our hospital with sudden onset of dyspnea. Prior to this event, the pleura was involved in an epithelial malignancy, which was immunohistochemically negatively stained with anti-D2-40, WT-1, or anti-calretinin antibodies, which are positive markers of mesothelioma. Transthoracic echocardiography revealed a fragile and mobile tumor occupying the right atrium, and the patient was admitted for surgical tumorectomy. The operation was performed urgently using a cardiopulmonary bypass via a full sternotomy. The pericardium is grossly intact and does not adhere to the heart. A 3 × 5 cm tumor was tightly attached to the right atrium and was large enough to fit into the tricuspid valve. Therefore, the entire margin of the tumor stem attachment was resected from the lateral wall of the right atrium. Although the resected tumor was not positive for any of the three histopathological markers of mesothelioma, *CDKN2A* co-deletion revealed by fluorescence in situ hybridization led to a diagnosis of malignant mesothelioma.

**CONCLUSIONS:**

Surgical removal of intracardiac tumors that cause circulatory and respiratory instability is essential for the prevention of sudden death, regardless of prognostic determinants. This case demonstrates that mesotheliomas can metastasize to the endocardium. Even when nuclear atypia and negative results for immunohistochemical tests for the three mesothelioma markers suggest carcinoma, mesothelioma should still be considered and *p16/CDKN2A* co-deletion should be evaluated.

## Abbreviations

BNP
: brain natriuretic peptide
DIC
: disseminated intravascular coagulation
ICU
: intensive care unit
FISH
: fluorescence in situ hybridization
PET/CT
: positron emission tomography/computed tomography
POD
: postoperative day

## INTRODUCTION

Pericardial mesothelioma accounts for the majority of primary pericardial tumors but is rare, with <50 cases of intracardiac involvement, according to a systematic review.^1)^ However, occasional reports have been published of pleural mesothelioma progressing from pericardial invasion into the myocardium, with the mode of pleural to intracardial extension generally being local infiltration.^2)^ Intracardiac metastases without pericardial invasion, as seen in the present case, are rare and unexpected. The surgical prognosis for intracardiac malignancies is very poor regardless of radical resection; however, the dilemma is about whether prompt surgical resection is essential to save the patient’s life.^3)^

## CASE PRESENTATION

**Fig. 1:**
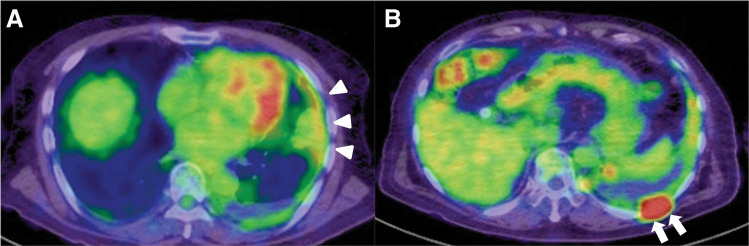
Positron emission tomography/computed tomography (PET-CT) performed 10 months prior to the emergent cardiac surgery. (**A**) Nodular-to-cordiform accumulation over a wide area of the left pleura (arrowheads). No obvious intra-atrial accumulation was observed. (**B**) Mass-like accumulation in the dorsal aspect of the left tenth intercostal space (arrows).

**Fig. 2:**
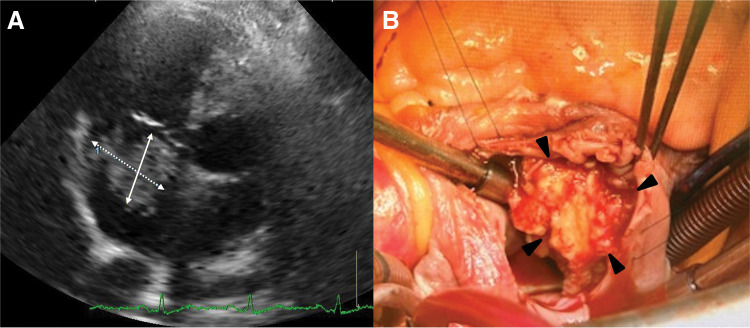
(**A**) Transthoracic echocardiography showing a 50 × 30 mm mass in the right atrium (arrows). (**B**) Intraoperative image showing tumor attached to the right atrial wall (arrow heads).

An 81-year-old man with a history of asbestos exposure presented to our hospital with sudden onset of dyspnea. Based on positron emission tomography/computed tomography (PET/CT) reports showing pleural accumulation (**Fig. 1**), a surgical pleural biopsy was performed 10 months prior to surgery. The patient was diagnosed with cancer at an unknown primary site, which was suspected to be a lung adenocarcinoma or malignant mesothelioma. He received seven courses of immune checkpoint inhibitor treatment, similar to the treatment for non-small cell lung cancer, until 6 months prior to surgery. He also had a history of hypertension, diabetes, epilepsy, unruptured brain aneurysms, and was undergoing treatment for prostate cancer. On examination, the patient was orthopedic, tachypneic, and hypoxic despite oxygen administration. Blood samples showed an elevated inflammatory response, anemia (hemoglobin: 9.8 g/dL and hematocrit: 31.9%), mild renal dysfunction (blood urea nitrogen: 31.1 mg/dL, creatinine: 0.99 mg/dL, and estimated glomerular filtration rate: 55.6 mL/min/1.73 m^2^), markedly elevated brain natriuretic peptide (BNP) (2480 pg/mL) and markers suggestive of disseminated intravascular coagulation (platelets: 3.8 × 10^4^/μL, prothrombin time: 16.5 s, fibrin/fibrinogen degradation products: 64 μg/mL, and D-dimer: 16.4 μg/mL). Transthoracic echocardiography revealed a fragile and mobile tumor occupying the right atrium (**Fig. 2A**), and the patient was admitted for surgical tumorectomy. Preoperative contrast-enhanced CT could not be performed because of a history of allergy to contrast media. The operation was performed urgently using a cardiopulmonary bypass via a full sternotomy. The pericardium is grossly intact and does not adhere to the heart. A 3 × 5 cm tumor was tightly attached to the right atrium and was large enough to easily fit into the tricuspid valve. Therefore, the entire margin of the tumor stem attachment was resected (**Fig. 2B**). The patient was transferred to the intensive care unit (ICU) for ventilatory management, was extubated on postoperative day (POD) 4, and discharged from the ICU on POD 17, followed by discharge on POD 54. Additional anticancer therapy was considered but was not suitable due to the patient’s physical condition being significantly reduced. Consequently, the patient died of pneumonia 2 months after surgery. Autopsies were not performed as the request of the family.

### Materials and Methods

#### Immunohistochemistry

Antibodies were purchased (maker and catalog number) and diluted as follows: AE1/3 (Agilent, Santa Clara, CA, USA, M3515, ×400), calretinin (Agilent, M7245 ×20), WT-1 (Leica Biosystems, Nussloch, Germany, CT-L-WT-1-562, ×30), CK7 (Agilent, M7018, ×500), CK20 (Agilent, M7019, ×900), desmin (Agilent, IS606, ×2), napsin A (Nichirei Biosciences, Tokyo, Japan, 438251, work solution), GATA-3 (Biocare Medical, Concord, CA, USA, ACR405A, ×100), D2-40/Podoplanin (Agilent, M3619, ×150), CEA (Agilent, A0115, ×30000), EMA (Agilent, M0613, ×1000), TTF-1 (Agilent, M3575, ×1000), and CK5/6 (Agilent, M7232, ×200). The antigen retrieval method was used to stain CK7 cells using proteinase K at 37°C for 5 min. Another method was used with other antibodies using Tris-EDTA (pH 9.0) at 100°C for 20 min.

#### FISH analysis

The FISH kit for *CDKN2A* (p16)/Ch-9 was purchased from Jokoh (Kawasaki, Japan; Catalog No. J17946).

#### Pathological Findings

**Fig. 3:**
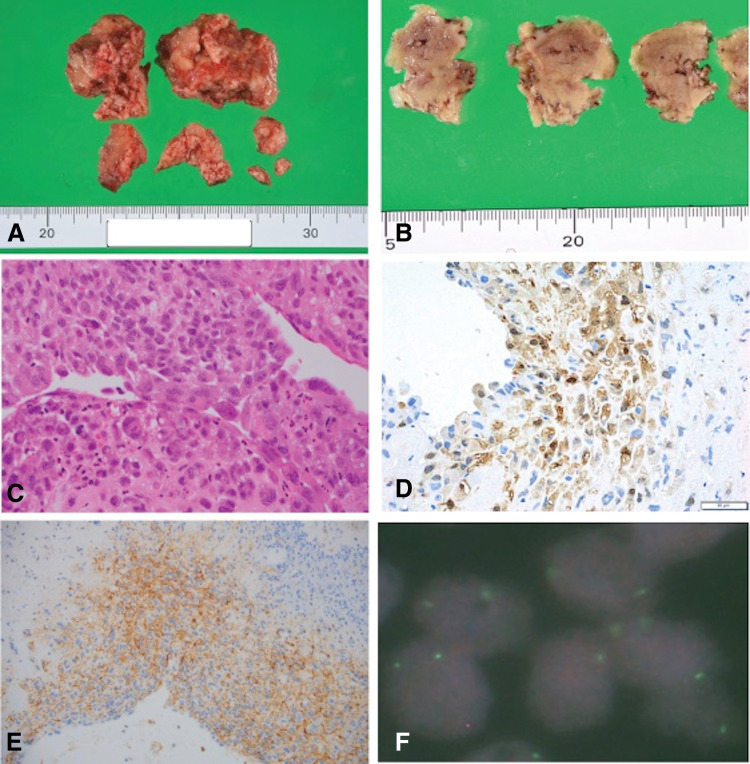
Photographs of excised cardiac tumor and microscopy images. (**A**) Macroscopically piece-meal resected tumor tissue is inconsistent and easily broken. (**B**) On cut surfaces of the fixed specimen show necrotic color. (**C**) Microscopically, it has only a small viable area composed of atypical cells with a high nucleus-to-cytoplasm ratio, rough chromatin, and loosely adhered epithelial-like features. (**D**) The tumor cells are positively stained immunohistochemically with anti-calretinin antibody, (**E**) and with anti-D2-40 antibody. (**F**) Fluorescence in situ hybridization of *CDKN2A* (*p16 INK4A*) shows an absent red-colored signal, which indicated co-deletion of *CDKN2A*, while blue signals were observed, indicating the chromosome 9 centromere.

Intraoperative findings revealed that the tumor was adhered to the right atrial free wall, 4.2 × 3.2 × 2.8 cm in size, yellow-light brown in color, with a rough and brittle surface, and was resected as one lump with the right atrial myocardium (**Figs. 3A**, **3B**). Histological examination revealed that the central part of the tumor was mostly composed of necrotic tissue, which was focally surrounded by a thin proliferation of tumor cells with eosinophilic cytoplasm, atypical nuclei, and acidophilic nucleoli (**Fig. 3C**). The histological findings were similar to those of the specimens obtained during pleural biopsy. Immunostaining of pleural biopsy specimens obtained 10 months prior to emergency cardiac surgery showed negative results for mesothelial-positive markers such as WT-1, calretinin, and D2-40 (**Figs. 3D**, **3E**). These immunohistological results, in addition to nuclear atypia, suggested the presence of a carcinoma rather than a mesothelioma.

After immunohistological examination of the resected specimen from the right atrium, we conducted FISH for *CDKN2A/p16* (indicated by red immunofluorescence) and chromosome 9 centromere (indicated by green immunofluorescence).^4)^ **Figure 3F** shows homozygous loss of *CDKN2A/p16*, suggestive of mesothelioma.

Cytological examination of the pericardial fluid revealed malignant findings. However, no gross findings suggestive of a pericardial tumor were noted.

## DISCUSSION

Based on Japanese demographic statistics, the number of mesothelioma-related deaths is increasing, and this trend is expected to continue in the foreseeable future.^5)^ Malignant mesothelioma continues to have a very poor prognosis owing to the difficulty in diagnosis and its rapid progression. However, recent developments in immune checkpoint inhibitor treatments are expected to improve the prognosis. There is an urgent need to enhance the accuracy of early diagnosis for prompt therapy.^6,7)^

The premise was that the patient underwent pleural biopsy 9 months prior to surgery for suspected malignant mesothelioma; however, a definitive diagnosis was not reached, and the patient received chemotherapy with pembrolizumab for non-small cell lung cancer. The pleural lesions shrank, and chemotherapy was discontinued 4 months after the start of treatment because of side effects. However, three CT scans before this episode showed no evidence of exacerbation. Additionally, an echocardiogram performed for screening before chemotherapy showed neither pericardial effusion nor an intracardiac mass.

The histopathological diagnosis of malignant mesothelioma was based on the confirmation of two positive mesothelial markers and two negative carcinoma markers by immunostaining. In the present case, the mesothelial markers calretinin, D2-40, and WT-1 did not show the characteristically positive staining for mesothelioma, whereas the cancer markers CEA, TTF-1, and Napsin A showed negative staining. Second, *CDKN2A/p16* co-deletion detected by FISH is thought to be useful in differentiating sarcomatoid mesothelioma from fibrotic pleurisy^4)^ and mesothelioma from reactive mesothelial hyperplasia. We analyzed *CDKN2A/p16* status in metastatic tumors and detected a clear homozygous deletion, which, combined with immunohistochemical analysis, indicated that the histological diagnosis was mesothelioma. These results were retrospectively consistent with mesothelioma as the diagnosis of pleural lesions. In the right atrium, mesothelial neoplasms do not originate as primary neoplasms. Therefore, this atrial tumor should be considered a metastatic neoplasm.

To the best of our knowledge, only three cases of malignant mesothelioma causing distant metastases to cardiac constituents, rather than direct invasion, have been reported. Senkottaiyan et al. reported a case of distant metastasis to the left ventricular outflow tract after resection of primary pleural mesothelioma of bilateral pleural origin. Tumorectomy was performed via left ventriculotomy, and no invasion of the myocardium was present; however, it soon recurred in the left atrium, resulting in the patient’s death within a year after surgery.^2)^ Kabbash et al. reported two cases of intracardiac metastases from peritoneal and pleural mesothelioma.^8)^ In the first case, 8 years after chemotherapy for peritoneal mesothelioma with multiorgan metastases, echocardiography revealed extensive intracardiac metastases, which caused dyspnea, orthopnea, and lower limb swelling. An intramyocardial tumor occupying the right ventricular apex, left ventricular anterolateral wall, and the anterior wall of the apex led to the patient’s death 5 months later. Another autopsy case, in which the patient died approximately 4 years after the initial treatment, including pleurectomy, showed a 7 mm-sized tumor within the myocardial septum, in addition to extensive metastases to other organs. In a previous report,^2)^ the tumor was attached to the endocardium, which may have been the same as in our case. In the latter report,^8)^ the tumor was an intramyocardial metastatic tumor, unlike in the present case.

Bussani et al. classified cardiac extension by mesothelioma as follows: (1) direct invasion, (2) metastasis through vessels, (3) metastasis through lymphatics, and (4) transluminal extension through the inferior vena cava or pulmonary veins.^9)^ Additionally, myocardial metastases are exclusively the result of retrograde lymphatic spread via tracheal or bronchomediastinal channels, whereas endocardial metastases are usually the result of infiltration into the ventricles from the bloodstream due to intraluminal loading, or secondary to diffusion from myocardial metastases. Additionally, endocardial metastases are usually localized to the right side of the heart and, although rare, are often associated with tumors with endovascular growth such as renal, liver, and uterine cancers. Jayaranagaiah et al. highlighted the usefulness of pericardial fluid cytology because the primary tumor and pericardial cavity are connected by a complex lymphatic network.^10)^ In this respect, venous return from the pleura may have led to metastasis to the right atrium.^11)^

Shenoy et al. described cases of circulatory failure in which a known or previously diagnosed cancer had progressed and required urgent treatment as a cardiovascular oncological emergency.^12)^ Depending on the tumor location, symptoms may present as embolization, obstruction, or arrhythmia. Obstruction of the atrioventricular valve by a tumor arising from the atrium can present with symptoms of dyspnea on exertion. In the present case there was a finding on echocardiography of the tumor showing a fit into the tricuspid valve which induced respiratory failure. Although the prognosis of secondary cardiac metastases remains unclear in cases of endocardial metastases, surgical resection should be considered to prevent or treat acute or complete obstruction. The patient in the present case was discharged postoperatively. However, the poor performance status made it difficult to treat him with anticancer drugs. The patient died of pneumonia 2 months postoperatively, and pathology, including that of an autopsy, was not performed and therefore no further data were acquired.

## CONCLUSIONS

Surgical removal of intracardiac tumors that cause circulatory and respiratory instability is essential for the prevention of sudden death, regardless of prognostic determinants. This case demonstrates that mesotheliomas can metastasize to the endocardium. Differentiating carcinoma from mesothelioma based on immunohistochemical results and FISH analysis of *CDKN2A/p16* co-deletion is important, leading to a better therapeutic strategy.

## ACKNOWLEDGMENTS

The authors would like to thank the technologists at the Division of Pathology of Nagano Red Cross Hospital, especially Ms. Chika Takeda, for their technical assistance and thank the advice from Editage (www.editage.com) for English language editing.

## DECLARATIONS

### Funding

There was no funding for this work.

### Authors’ contributions

The contributions of each author are as follows:

TT and II wrote most of the manuscript.

II suggested the context of the manuscript.

TT, MY, KT, YM, and TK cared for the patient and suggested the content of the manuscript.

SS conducted immunohistochemistry and examined the cytological specimens.

All the authors have read and approved the manuscript.

### Availability of data and materials

All related data are included within the article.

### Ethics approval and consent to participate

This study was conducted according to the principles of the Declaration of Helsinki. This work does not require ethical considerations or approval.

### Consent for publication

Informed consent was obtained from the patient for publication of the patient’s clinical data and accompanying images.

### Competing interests

The authors declare that they have no competing interests.
